# Statistics about torrents in Lower Austria, status from May 2015

**DOI:** 10.1016/j.dib.2015.07.036

**Published:** 2015-08-07

**Authors:** Ehrenfried Lepuschitz

**Affiliations:** Institute of Mountain Risk Engineering at the University of Natural Resources and Life Sciences, Vienna

## Abstract

This data presents analyzed data exports of Austrian torrent and avalanche cadaster (TAC) in May 2015. The TAC is developed by Austrian Service for Torrent and Avalanche Control. Data are viewed from different aspects and combinations geographically in the area of Lower Austria, a province of Austria.

## 1 Specifications Table

**Table:** 

Subject area	Statistics, Mountain Risk and Disaster Management, Torrent and avalanche cadaster (TAC)
More specific subject area	Torrents, Hazard zones
Type of data	Text, tables, figures
How data was acquired	Geographic information system (GIS), TAC
Data format	Raw and analyzed
Experimental factors	TAC is developed since 2004. In Lower Austria since 2010 torrent catchment areas and hazard zone maps are digitized in TAC. Quantity and quality of digitized data are reaching good levels for analyzing data in different points of view. Data statistics in this data paper were made in May 2015
Experimental features	In GIS torrent catchment areas and hazard zone maps are digitized and analyzed. Data were summarized for counties and the whole province of Lower Austria
Data source location	Vienna, Austria, Europe
Data accessibility	Data are included in this paper

## 2 Value of the data

• In Austrian water bodies exists a separation in torrents and rivers based on federal law [1]. This situation can be discussed with given statistics in this data paper.
• The paper shows also the dispersion and amount of torrent catchment areas and hazard zone maps in counties and the province of Lower Austria.
• The dispersion of hazard zones caused by torrents can be used for future strategies for preventions. Settlements are often build in menaced locations how should this be managed in the future.

## 3 Methods

**Fig. 1:**
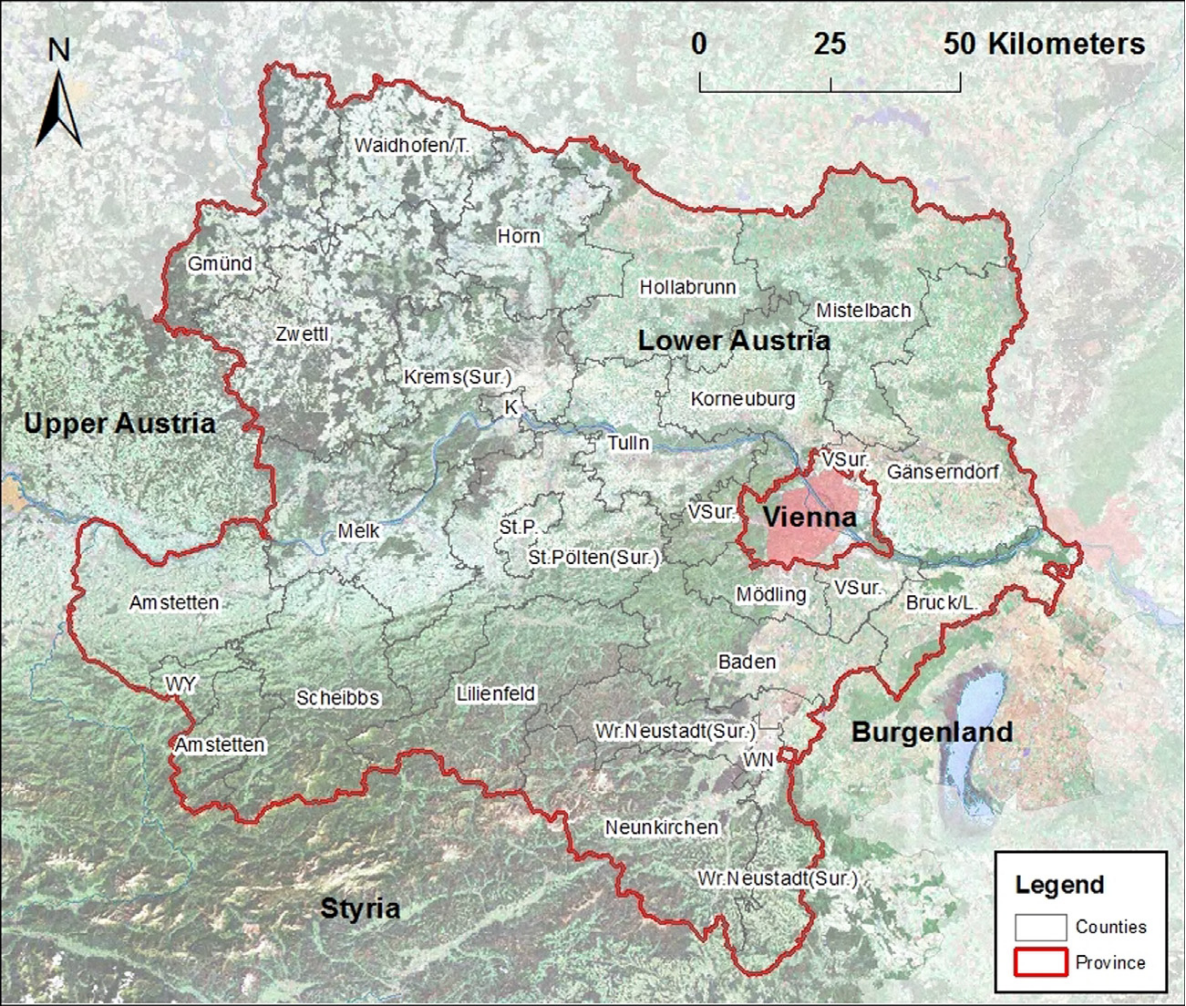
Counties and cities with own statute in Lower Austria; K=Krems (city), L=Leitha, St. P.=St. Pölten (city), Sur.=surroundings, T=Thaya, VSur.=Vienna-Surroundings, WN=Wr. Neustadt (city), WY=Waidhofen/Ybbs (city), TAC 2015.

The analyzed data presented here were exported of Austrian TAC (see Chapters 2 and 3 in [1]) with the focus on Austrian federal province Lower Austria. Since 2011 a lot of data were digitized in TAC in Lower Austria. Lower Austria is divided into 21 counties and four cities with own statute, each county has different number of villages. Fig. 1 shows the fragmentation of Lower Austria in its counties and cities with own statute. Each village and city has its own municipality government. Cities with own statute are treated like counties in the statistics.

## 4 Experimental design and data

### 4.1 Torrent catchment areas

**Fig. 2:**
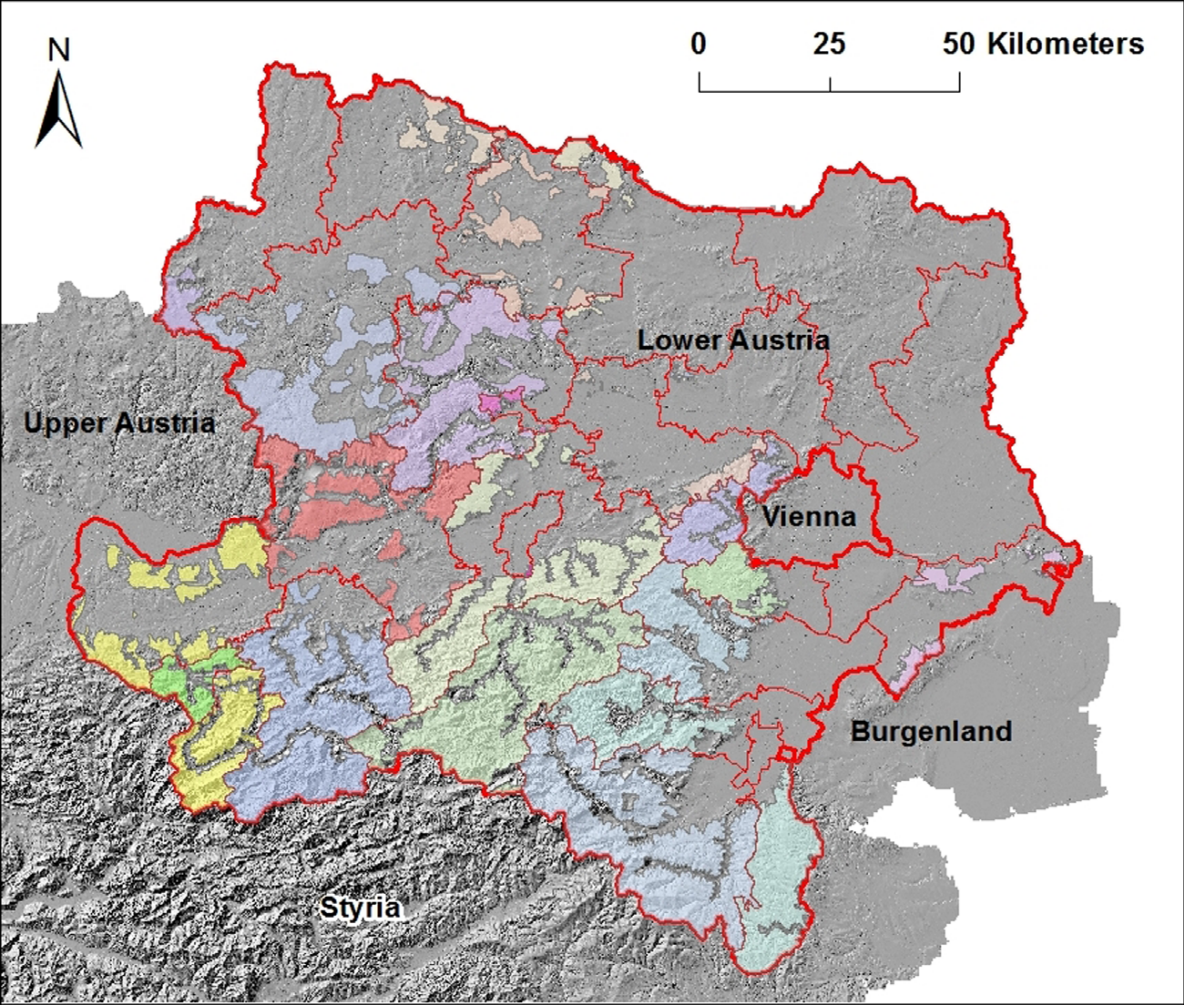
Merged torrent catchment areas above hillshade layer, the torrent catchment areas are merged and colored per county; some counties in the northeast do not have torrents, along the border to Styria counties have the most coverage of torrent catchment areas in Lower Austria, TAC 2015.

In Austria torrents are defined in Austrian Forest Act 1975 [2]. Torrents exist only in mountainous regions. The hillshade layer in Fig. 2 is covered by torrent catchment areas in mountainous areas, and lowlands are not covered. Counties and cities have different coverage of torrent catchment areas.

**Fig. 3:**
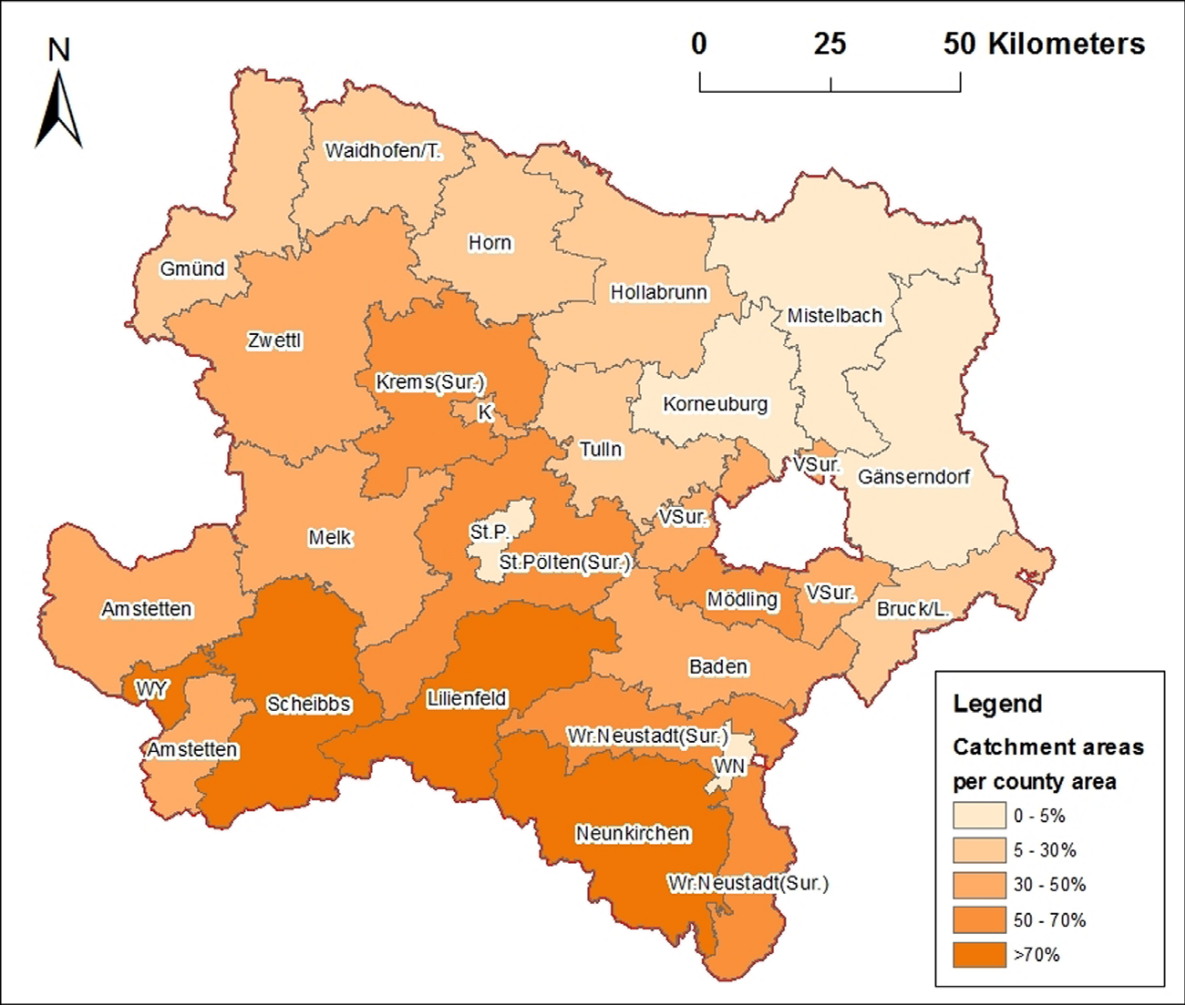
%-Coverage of torrent catchment areas per county; K=Krems (city), L=Leitha, St. P.=St. Pólten (city), Sur.=Surroundings, T=Thaya, VSur.=Vienna-Surroundings, WN=Wr. Neustadt (city), WY=Waidhofen/Ybbs (city), TAC 2015.

In the whole province of Lower Austria torrent catchment areas cover around 7000 ${\mathrm{km}}^{2}$ or 36% of the province area. In Table 1 and Fig. 3 torrent catchment area coverage per county is shown.

### 4.2 Hazard zones of torrents

Since 1976 in Austria a federal regulation defines the structure and development process of hazard zone maps in torrent and avalanche areas [3]. In the following statistics only torrents and their hazard zones in Lower Austria are included.

**Fig. 4:**
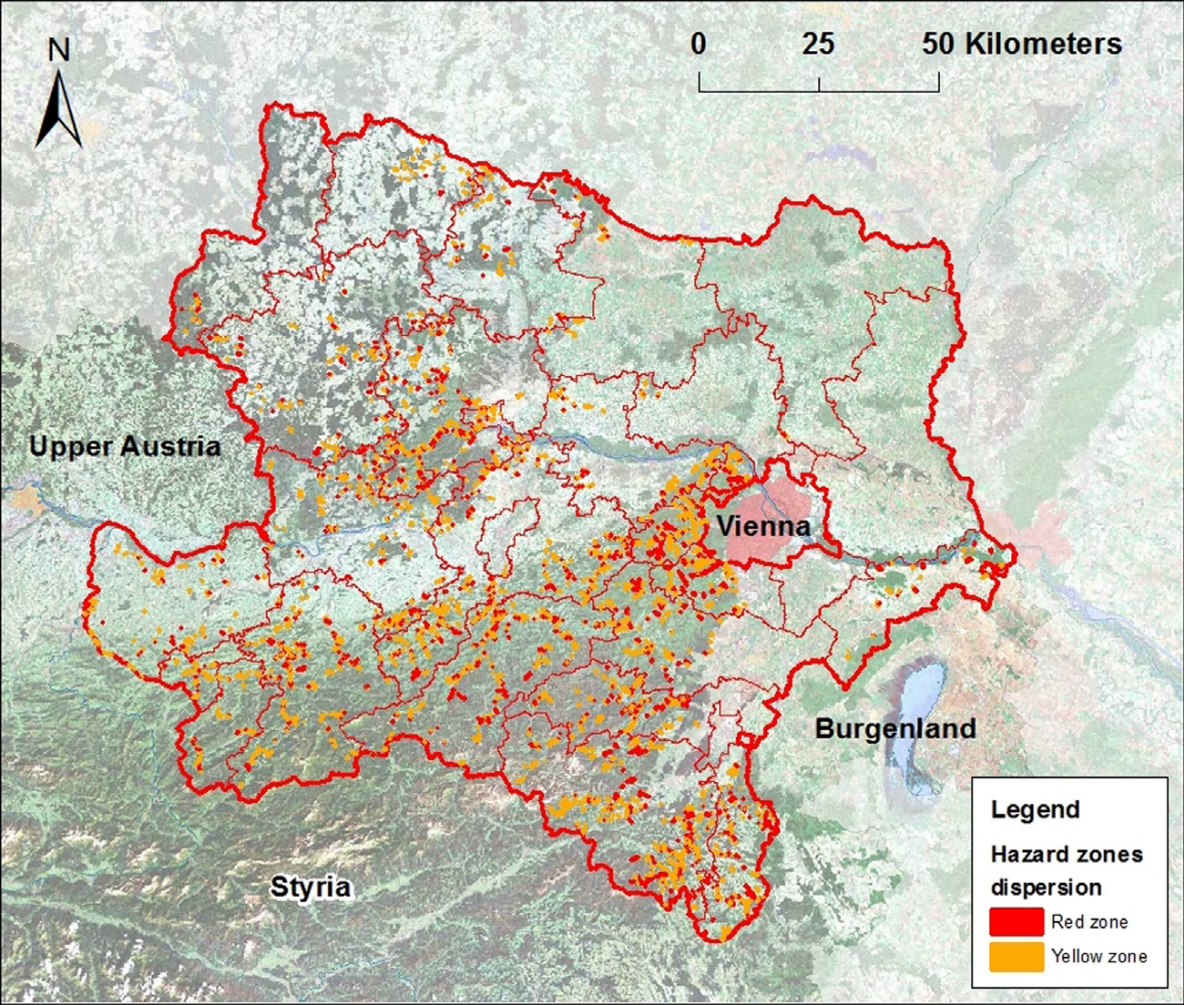
Dispersion of yellow and red hazard zones of torrents in Lower Austria, TAC 2015.

Hazard zones of torrents are developed only in settlements, Fig. 4 shows the dispersion in whole Lower Austria. Hazard zones also demonstrate where torrents flow through settlements. West of Vienna more hazard zones were developed which illustrate that there are more and bigger settlements in mountainous regions. In lowlands there are no torrents and close to the Styrian border are mountainous regions without or only small settlements and therefore less hazard zones.

**Fig. 5:**
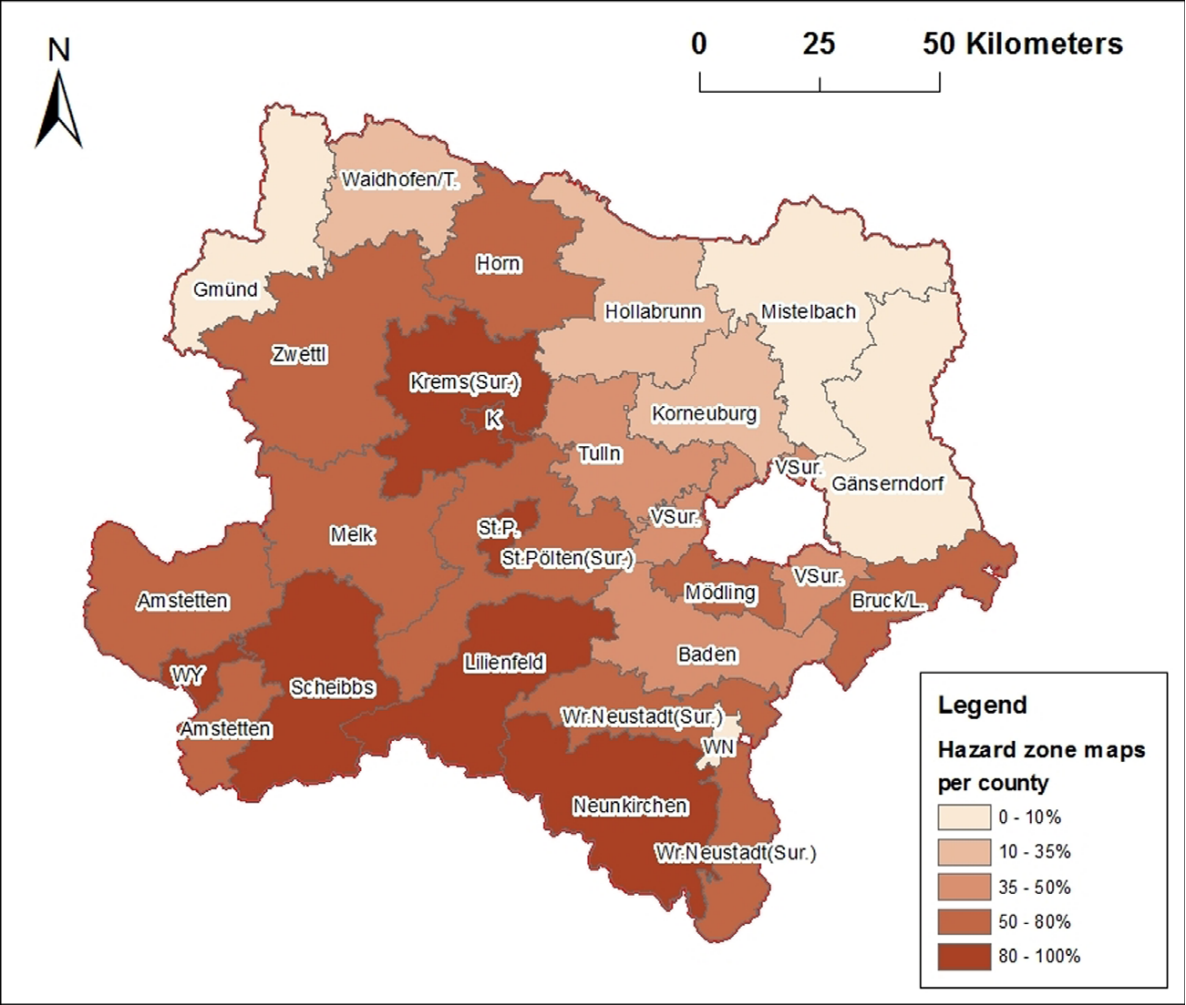
%-Villages with hazard zone maps per county; K=Krems (city), L=Leitha, St. P.=St. Pölten (city), Sur.=Surroundings, T=Thaya, VSur.=Vienna-Surroundings, WN=Wr. Neustadt (city), WY=Waidhofen/Ybbs (city), TAC 2015.

Austrian federal regulation for hazard zone maps defines that for each village with torrents in settlements a hazard zone map has to be developed. Villages which have torrent catchment areas outside of settlements do not get hazard zone maps. Table 2 and Fig. 5 demonstrate how many villages are part of the counties and how many of them have hazard zone maps.

Three cities with own statute (Krems, St. Pölten and Waidhofen/Ybbs) have hazard zone maps (100%), Wr. Neustadt (city) has no torrents therefore no hazard zone map is developed. Summarized, in Lower Austria around 53% of its villages and cities own hazard zone maps.

### 4.3 Comparison

Comparisons of Tables 1 and 2 or Figs. 3 and 5 show that the counties of Lilienfeld, Neunkirchen and Scheibbs have the most coverage of torrent catchment areas and the most villages with hazard zone maps. Gänserndorf and Mistelbach counties are free of torrent hazards. In some counties like Krems (Sur.), Horn, etc., the percentage of hazard zone maps is higher than the percentage of the coverage of torrent catchment areas per county. Also in whole Lower Austria around 53% of its villages and cities own a hazard zone map but only 36% of the province area is covered by torrent catchment areas.

## Figures and Tables

**Table 1:** Torrent catchment (TC) areas per counties, absolute and relative to the county area. Finally also for whole Lower Austria. Sur.=Surroundings; TAC 2015.

County	County area $\left({\mathrm{km}}^{2}\right)$	TC-area $\left({\mathrm{km}}^{2}\right)$	% TC-area per county
Amstetten	1186.2	551.7	47
Baden	753.6	325.9	43
Bruck/Leitha	495.0	88.6	18
Gänserndorf	1272.0	0.0	0
Gmünd	786.7	63.5	8
Hollabrunn	1010.8	65.3	6
Horn	783.7	173.8	22
Korneuburg	626.8	4.6	1
Krems (city)	51.7	23.2	45
Krems (Sur.)	922.9	508.9	55
Lilienfeld	932.1	783.5	84
Melk	1014.3	445.7	44
Mistelbach	1292.5	0.5	0
Mödling	277.5	143.1	52
Neunkirchen	1150.2	851.3	74
St. Pölten (Sur.)	1122.6	594.0	53
St. Pölten (city)	108.4	2.1	2
Scheibbs	1023.7	734.6	72
Tulln	657.8	77.9	12
Waidhofen/Thaya	669.1	85.0	13
Waidhofen/Ybbs (city)	131.2	108.3	83
Wr. Neustadt (Sur.)	972.3	644.0	66
Wr. Neustadt (city)	60.9	0.0	0
Vienna-Sur.	485	190.1	39
Zwettl	1399.1	502.2	36

Lower Austria	19 186.1	6967.8	36

**Table 2:** Hazard zone (HZ) maps per counties, absolute and relative to the amount of villages per county. City counties have only one village (0% or 100%). Sur.=Surroundings; TAC 2015.

County	Villages per county	Villages with HZ maps	%-Villages with HZ maps
Amstetten	34	25	73.5
Baden	30	13	43.3
Bruck/Leitha	20	15	75.0
Gänserndorf	44	0	0.0
Gmünd	21	2	9.5
Hollabrunn	24	5	20.8
Horn	20	12	60.0
Korneuburg	19	2	10.5
Krems (city)	1	1	100.0
Krems (Sur.)	30	24	80.0
Lilienfeld	14	14	100.0
Melk	40	28	70.0
Mistelbach	36	0	0.0
Mödling	20	13	65.0
Neunkirchen	44	36	81.8
St. Pölten (Sur.)	39	31	79.5
St. Pölten (city)	1	1	100.0
Scheibbs	18	17	94.4
Tulln	21	10	47.6
Waidhofen/Thaya	15	2	13.3
Waidhofen/Ybbs (city)	1	1	100.0
Wr. Neustadt (Sur.)	35	25	71.4
Wr. Neustadt (city)	1	0	0.0
Vienna-Sur.	21	8	38.1
Zwettl	24	17	70.8

Lower Austria	573	302	52.7
